# Renal histopathological predictors of end-stage kidney disease in ANCA-associated vasculitis with glomerulonephritis: a single-centre study in Korea

**DOI:** 10.1038/s41598-023-41811-0

**Published:** 2023-09-08

**Authors:** Sung-Eun Choi, Soo Bin Lee, Jung Yoon Pyo, Sung Soo Ahn, Jason Jungsik Song, Yong-Beom Park, Beom Jin Lim, Sang-Won Lee

**Affiliations:** 1 Department of Pathology, CHA University, CHA Bundang Medical Center, Seongnam-si, Kyeonggi-do Republic of Korea; 2 Department of Medicine, Yonsei University College of Medicine, Seoul, Republic of Korea; 3 Division of Rheumatology Department of Internal Medicine, Yonsei University College of Medicine, 50-1 Yonsei-ro, Seodaemun-gu, Seoul, 03722 Republic of Korea; 4 Institute for Immunology and Immunological Diseases, Yonsei University College of Medicine, Seoul, Republic of Korea; 5 Department of Pathology, Gangnam Severance Hospital, Yonsei University College of Medicine, 211 Eonju-ro, Gangnam-gu, Seoul, 06273 Republic of Korea

**Keywords:** Membranoproliferative glomerulonephritis, Interstitial nephritis

## Abstract

This study investigated whether histopathological classification and histologic lesion scores could significantly and independently predict the progression to end-stage kidney disease (ESKD) in Korean patients with antineutrophil cytoplasmic antibody (ANCA)-associated vasculitis-glomerulonephritis (AAV-GN). This study included 113 patients with AAV-GN confirmed by kidney biopsy. The glomerular, tubulointerstitial, and vascular lesions were systematically assessed using a scoring system. The scoring system was adopted from the Banff scoring system but also the Oxford study and the revision of the ISN/RPS. For comparison, the scores were classified into two groups; the low, and the high, and the difference was investigated between ESKD and non-ESKD groups using Cox proportional analysis. At diagnosis, the median age was 59.0 years and 33.6% were males. Of 113 patients, 44.2% had ESKD progression during follow-up. There were significant differences in several kidney-, inflammation-, and AAV-pathogenesis-related variables between AAV-GN patients with ESKD and those without. The sclerotic class exhibited the worst renal prognosis among the four histopathological classes. Among histopathological features, high interstitial fibrosis, tubular atrophy and global glomerulitis scores were significantly associated with ESKD progression. Whereas multivariable Cox analysis revealed only a high global glomerulitis score which means global endocapillary hypercellularity in a larger number of glomeruli is an independent predictor of ESKD progression. Moreover, among clinical and histopathological features, a high global glomerulitis score could also predict ESKD progression in addition to serum blood urea nitrogen and creatinine. This study demonstrated the worst renal prognosis for the sclerotic class and first discovered that a high global glomerulitis score was an independent predictor of ESKD in patients with AAV-GN.

## Introduction

Antineutrophil cytoplasmic antibody (ANCA)-associated vasculitis (AAV), one of two types of small vessel vasculitis, is characterised by necrotising vasculitis occurring primarily in capillaries, arterioles, and venules, and occasionally in medium-sized arteries^1^. AAV has three traditional subtypes according to clinical, laboratory, radiological, and histological features: eosinophilic granulomatosis with polyangiitis (EGPA), granulomatosis with polyangiitis (GPA), and microscopic polyangiitis (MPA)^1–3^. The role of ANCA in the pathogenesis of AAV has been described; given differences in the genetic background or clinical expression based on ANCA type, AAV may be divided into three subtypes: myeloperoxidase (MPO)-ANCA vasculitis, proteinase 3 (PR3)-ANCA vasculitis, and ANCA-negative vasculitis^4^. In addition, the histopathological pattern of glomerulonephritis (GN) differs between MPO-ANCA and PR3-ANCA vasculitis^5^.

Among the three traditional AAV subtypes, the reported frequencies of renal involvement were up to 90% in patients with MPA, 50–80% in those with GPA, and 4–51% in those with EGPA^6^. A recent study reported that renal involvement was the most common manifestation, with a reported frequency of 58.2% in 182 Korean patients with AAV^7^. Another recent study observed a 27.8% frequency of end-stage kidney disease (ESKD) in Korean patients with AAV^8^. To date, several baseline risk factors for the progression to ESKD in patients with AAV have been reported including age, serum creatinine, estimated glomerular filtration rate (eGFR), blood pressure, the proportion of abnormal glomeruli, tubular atrophy, interstitial fibrosis on kidney biopsy^9–11^.

In 2010, Berden et al. categorised ANCA-associated vasculitis and GN (AAV-GN) into four classes based on histopathological features: (i) focal class: ≥ 50% normal glomeruli; (ii) crescentic class: ≥ 50% glomeruli with cellular crescents; (iii) mixed class: < 50% normal, < 50% crescentic, and < 50% globally sclerotic glomeruli; and (iv) sclerotic class: ≥ 50% globally sclerotic glomeruli. They also demonstrated higher kidney survival rates in the order of focal, crescentic, mixed, and sclerotic classes^12^. A recent study provided information on the predictive potential for kidney outcomes of both histopathological and clinicopathologic classifications in Korean patients with AAV-GN^13^. However, the study had three limitations. First, no classification of AAV subtypes was performed in the two methods, the traditional subtypes and ANCA-based subtypes. Second, the multivariable analysis did not include MPO-ANCA (or perinuclear (P)-ANCA) and/or PR3-ANCA (or cytoplasmic (C)-ANCA) which are known to be associated with poor kidney outcomes. Third, the histological lesion scores were not described in detail. To overcome these issues, the present study included variables related to the five AAV-GN subtypes including EGPA-GN, GPA-GN, MPA-GN, renal limited vasculitis (RLV) and unclassifiable pauci-immune GN (UPIGN), and analysed their detailed histomorphologic features by adopting well-established histological parameters used in other kidney diseases. In addition to the ANCA type, this study also investigated whether histopathological classification and histologic lesion scores could significantly and independently predict the progression to ESKD in Korean patients with AAV-GN.

## Patients and methods

### Patients

This study retrospectively screened the medical records of 126 patients with AAV-GN, including five subtypes: EGPA-GN, GPA-GN, MPA-GN, RLV, and UPIGN. In patients who underwent kidney biopsy, RLV was defined as AAV with only crescentic glomerulonephritis and without any further involvement of major organs^14^. Conversely, in patients not undergoing kidney biopsy, RLV was classified in patients fulfilling all four requirements: (i) no kidney biopsy, (ii) no surrogate marker suggesting GPA, (iii) the presence of ANCA and (iv) red blood cell (RBC) cast-related haematuria or > 10% dysmorphic RBC or 2 + haematuria and 2 + proteinuria on urine sticks according to the 2007 European Medicine Agency (EMA) algorithm for AAV^2^. UPIGN was defined as AAV-GN exhibiting histopathological crescent formation with no evidence of immune deposits and was not further classified as EGPA-GN, GPA-GN, MPA-GN or RLV^15^. The inclusion criteria were (i) patients who fulfilled both the 2012 revised International Chapel Hill Consensus Conference Nomenclature of Vasculitides and the 2007 EMA algorithm for AAV and renal vasculitis^1,2^; (ii) patients first diagnosed with AAV and AAV-GN at the Division of Rheumatology, Department of Internal Medicine, Yonsei University College of Medicine, Severance Hospital, from April 2006 to March 2019 based on the day on which the kidney biopsy performed; (iii) patients with well-documented medical records sufficient to collect clinical and laboratory data including ANCA results, calculate Birmingham vasculitis activity score and five-factor score at AAV diagnosis, and assess the development of ESKD and all-cause mortality during follow-up; (iv) patients who underwent kidney biopsy and had histopathological results;, and (v) patients who were followed up for at least 6 months from the diagnosis of AAV-GN. Meanwhile, the exclusion criteria were (i) patients with concomitant serious medical conditions that might confound the interpretation of the results, such as malignancies, hospitalised infectious diseases, and systemic vasculitides other than AAV or AAV-GN; and (ii) patients previously exposed to immunosuppressants for the treatment of AAV or AAV-GN before the diagnosis. Concomitant serious medical conditions and immunosuppressive drug administration were identified accounting to the 10th revised International Classification of Diseases and the Korean Drug Utilization Review system, respectively. Based on the inclusion and exclusion criteria, this study analysed the medical records of 113 patients with AAV-GN.

The present study was approved by the Institutional Review Board (IRB) of Severance Hospital (Seoul, Korea, IRB No. 4-2020-1071) and was conducted according to the tenets of the Declaration of Helsinki. Given the retrospective study design and the use of anonymised patient data, the requirement for written informed consent was waived by the Institutional Review Board (IRB) of Severance Hospital.

### Clinical and laboratory data

Variables regarding demographic, AAV-specific serum immunoglobulins and laboratory data, described in Table 1, were collected. In this study, all-cause mortality was defined as death due to any aetiology. ESKD was defined as the initiation of dialysis due to kidney function, indicated by eGFR < 15 mL/min/1.73 m^2^ and related uremic symptoms, after excluding acute kidney injury requiring dialysis^16^. For patients who progressed to ESKD, the follow-up duration based on ESKD was defined as the period between AAV-GN diagnosis based on kidney biopsy and ESKD occurrence. Conversely, for patients without ESKD, the follow-up duration was defined as the period between AAV-GN diagnosis and the last visit. The follow-up duration based on all-cause mortality was defined as the period between AAV-GN diagnosis and the last visit for surviving patients. For deceased patients, it was defined as the period between AAV-GN diagnosis and death. The number of patients who received glucocorticoids and immunosuppressive drugs administered after diagnosis was counted.

**Table 1:** Baseline characteristics of 113 patients with AAV-GN and comparison of variables between patients with ESKD and those without ESKD.

Variables	Values	Patients without ESKD (N = 63)	Patients with ESKD (N = 50)	P value
At AAV-GN diagnosis by kidney biopsy				
Demographic data				
Age (years)	59.0 (16.0)	62.0 (20.0)	58.5 (13.0)	0.561
Male gender (N, (%))	38 (33.6)	18 (28.6)	20 (40.0)	0.202
AAV-GN Subtypes (N, (%))				
EGPA-GN	5 (4.4)	4 (6.3)	1 (2.0)	0.155
GPA-GN	8 (7.1)	3 (4.8)	5 (10.0)	
MPA-GN	41 (36.3)	28 (44.4)	13 (26.0)	
RLV	33 (29.2)	15 (23.8)	18 (36.0)	
UPIGN	26 (23.0)	13 (20.6)	13 (26.0)	
ANCA positivity (N, (%))				
MPO-ANCA (or P-ANCA) positivity	81 (71.7)	48 (76.2)	33 (66.0)	0.232
PR3-ANCA (or C-ANCA) positivity	10 (8.8)	3 (4.8)	7 (14.0)	0.086
Both ANCA positivity	3 (2.7)	1 (1.6)	2 (4.0)	0.428
ANCA negativity	25 (22.1)	13 (20.6)	12 (24.0)	0.669
Routine Laboratory results				
White blood cell count (/mm^3^)	7620.0 (4455.0)	8250.0 (4540.0)	7430.0 (4465.0)	0.493
Neutrophil count (/mm^3^)	5440.0 (4280.0)	5720.0 (4250.0)	5220.0 (4535.0)	0.699
Lymphocyte count (/mm^3^)	1230.0 (870.0)	1220.0 (820.0)	1255.0 (837.5)	0.437
Eosinophil count (/mm^3^)	180.0 (235.0)	180.0 (260.0)	205.0 (232.5)	0.797
Haemoglobin (g/dL)	8.6 (1.9)	9.1 (1.9)	8.3 (1.9)	0.043
Platelet count (× 10^9^/L)	272.0 (159.0)	270.0 (170.0)	276.0 (137.5)	0.619
Fasting glucose (mg/dL)	93.0 (29.0)	95.0 (37.5)	91.0 (24.0)	0.102
BUN (mg/dL)	38.9 (33.7)	33.8 (24.6)	44.5 (32.2)	0.001
Serum creatinine (mg/dL)	3.0 (3.7)	2.3 (1.7)	5.3 (2.7)	< 0.001
eGFR (mL/min/1.73 m^2^)	16.0 (23.3)	23.0 (29.5)	10.0 (10.7)	< 0.001
Total cholesterol (mg/dL)	155.0 (51.5)	152.5 (56.8)	156.0 (50.5)	0.626
Uric acid (mg/dL)	5.9 (2.8)	5.6 (2.6)	6.5 (2.8)	0.005
Serum protein (g/dL)	6.1 (1.0)	6.2 (0.9)	6.1 (1.1)	0.201
Serum albumin (g/dL)	3.0 (8.0)	3.0 (0.8)	3.0 (0.9)	0.289
Acute phase reactants				
ESR (mm/h)	77.5 (61.3)	81.5 (62.0)	72.5 (66.3)	0.190
CRP (mg/L)	10.7 (50.9)	20.1 (79.9)	9.8 (28.5)	0.045
Urinalysis results				
Urine protein-to-creatinine ratio	2.3 (2.9)	1.9 (2.2)	3.6 (3.7)	0.002
Haematuria (N, (%))	90 (79.6)	50 (79.4)	40 (80.0)	0.934
Other results				
Serum IgA	259.0 (149.5)	261.0 (146.8)	239.0 (174.0)	0.752
C3 (mg/dL)	104.3 (39.6)	110.3 (40.2)	99.8 (24.1)	0.020
C4 (mg/dL)	27.7 (12.6)	27.9 (11.6)	27.7 (16.1)	0.701
During follow-up				
Poor prognosis				
ESKD (N, (%))	50 (44.2)	0 (0)	50 (100)	N/A
Follow-up period based on ESKD (months)	41.6 (73.5)	64.8 (58.8)	1.7 (26.1)	< 0.001
All-cause mortality (N, (%))	23 (20.4)	9 (14.3)	14 (28.0)	0.072
Follow-up period based on mortality (months)	64.1 (63.1)	56.4 (55.6)	67.2 (69.8)	0.324
Medications administered (N, (%))				
Glucocorticoids	113 (100)	63 (100)	50 (100)	N/A
Cyclophosphamide	47 (41.6)	29 (46.0)	18 (36.0)	0.283
Rituximab	19 (16.8)	13 (20.6)	6 (12.0)	0.223
Azathioprine	56 (49.6)	33 (52.4)	23 (46.0)	0.500
Mycophenolate mofetil	32 (28.3)	16 (25.4)	16 (32.0)	0.439
Tacrolimus	7 (6.2)	5 (7.9)	2 (4.0)	0.389
Methotrexate	3 (2.7)	1 (1.6)	2 (4.0)	0.428

Values are expressed as a median (interquartile range, IQR) or N (%).

*AAV* ANCA-associated vasculitis, *ANCA* antineutrophil cytoplasmic antibody, *EGPA* eosinophilic granulomatosis with polyangiitis, *GPA* granulomatosis with polyangiitis, *MPA* microscopic polyangiitis, *RLV* renal limited vasculitis, *GN* glomerulonephritis, *MPO* myeloperoxidase, *P* perinuclear, *PR3* proteinase 3, *C* cytoplasmic, *BUN* blood urea nitrogen, *eGFR* estimated glomerular filtration rate, *INR* international normalised ratio, *ESR* erythrocyte sedimentation rate, *CRP* C-reactive protein, *C3* complement 3, *C4* complement 4, *ESKD* end-stage kidney disease, *N/A* not applicable.

### Histopathological data

Two renal pathologists, B.J.L. and S.C., reviewed the kidney biopsy slides and performed scoring. Glomerular lesions were classified according to the presence of sclerosis, the extent of global and/or segmental sclerosis, the presence of the crescent, the proportion of cellular, fibrocellular, and fibrous crescents, and the presence of fibroid necrosis. Tubulointerstitial lesions were classified as acute tubular injuries or medullary angiitis. The presence or absence of each component was recorded. Vascular lesions were classified as arterial intimal fibrosis or arterial medial sclerosis, and the presence or absence of the lesions was recorded. The definitions of the crescent and glomerular fibrinoid necrosis were described in the revision of the ISN/RPS for lupus nephritis^17^. We also adopted the definition of the lesions and/or their scoring system from the Banff classification of renal allograft pathology and the Oxford classification of IgA nephropathy^18^. Mesangial hypercellularity was scored according to the definitions from the Oxford study^19^. Banff defines glomerulitis as “complete or partial occlusion of 1 or more glomerular capillaries by leukocyte infiltration and endothelial cell enlargement”. In addition, we devised another item, global glomerulitis, defined as “complete or partial occlusion of glomerular capillaries by leukocyte infiltration and endothelial cell enlargement involving nearly all the capillary loops of a glomerulus. Global glomerulitis was scored according to the proportion of glomeruli showing global glomerulitis; global glomerulitis in less than 25% of glomeruli as 1, global glomerulitis in 25–75% as 2, and global glomerulitis in more than 75% as 3 (Supplementary Methods S1). To compare the differences in scores between ESKD and non-ESKD groups, the grades were classified as ‘low’ (grades 0 and 1) and ‘high’ (grades 2 and 3). However, as the scores for cg were only 0 and 1, these grades were classified as ‘low’ and ‘high’, respectively. For global glomerulitis and glomerular fibrinoid necrosis, grade 0 (the absence of these conditions) as ‘low’ and grade 1 or more, that is, 1, 2, and 3(the presence of these conditions), as ‘high’, as it yielded the lowest p-values in the statistical analyses.

### Statistical analyses

All statistical analyses were performed using IBM SPSS Statistics for Windows, version 26.0 (IBM Corp., Armonk, NY, USA). Continuous variables were expressed as medians with interquartile ranges, whereas categorical variables were expressed as numbers (percentages). Significant differences between two categorical variables were analysed using the chi-square and Fisher’s exact tests. The Mann–Whitney U test was used to identify significant differences between two continuous variables. Comparisons of the cumulative ESKD-free and patients’ survival rates between the two groups were analysed using the Kaplan Meier survival analysis with log-rank tests. Since six times of comparisons of the cumulative ESKD-free survival rates were performed for the groups according to histopathological classification, statistical significance was set as P < 0.008 based on the Bonferroni’s method. The multivariable Cox hazard model using variables with statistical significance in the univariable Cox hazard model was used to obtain hazard ratios (HRs) during the considerable follow-up duration. Statistical significance was set as P < 0.05.

### Ethics approval and consent to participate

The present study was approved by the Institutional Review Board (IRB) of Severance Hospital (Seoul, Korea, IRB No. 4-2020-1071), and conducted according to the Declaration of Helsinki. Given the retrospective design of the study and the use of anonymised patient data, the requirement for written informed consent was waived.

## Results

### Baseline characteristics of patients with AAV-GN

At AAV-GN diagnosis, the median age was 59.0 years and 33.6% of the patients were male. Of the 113 patients with AAV-GN, 41, 33, 26, 8, and 5 had MPA-GN, RLV, UPIGN, GPA-GN and EGPA-GN, respectively. The detection rate of MPO-ANCA (or P-ANCA) was much higher than that of PR3-ANCA (or C-ANCA) (71.7% vs. 8.8%). Additionally, ANCA was not detected in one of the 33 patients with RLV and 24 of the 26 patients with UPIGN. The median serum blood urea nitrogen (BUN) level, serum creatinine level, eGFR and random urine protein-to-creatinine ratio were 38.9 mg/dL, 3.0 mg/dL, 16.0 mL/min/1.73 m^2^, and 2.3, respectively. The mean number of glomeruli per biopsy was 15.1062, and the median was 14. During follow-up, 50 patients (44.2%) exhibited the progression to ESKD for a median follow-up period of 41.6 months, and 23 patients (20.4%) died for a median period of 64.1 months (Table 1).

### Cross-sectional comparisons of variables at AAV-GN diagnosis between patients with ESKD and without ESKD

Herein, we selectively described the variables that showed statistically significant differences. At diagnosis, demographic data, AAV-GN subtypes and ANCA positivity did not differ between the two groups. Among laboratory results, patients with ESKD exhibited higher serum BUN (44.5 vs. 33.8 mg/dL, P = 0.001), creatinine (5.3 vs. 2.3 mg/dL, P < 0.001) and uric acid (6.5 vs. 5.6 mg/dL, P = 0.005) levels, and lower haemoglobin (8.3 vs. 9.1 g/dL, P = 0.043) and eGFR (10.0 vs. 23.0 mL/min/1.73m^2^, P < 0.001) values than those without ESKD. Patients with ESKD showed significantly higher proteinuria compared to those without ESKD (3.6 vs. 1.9, P = 0.002). Conversely, CRP (9.8 vs. 20.1 mg/L, P = 0.045) and C3 (99.8 vs. 110.3 mg/dL, P = 0.020) levels in patients with ESKD were lower than those in patients without ESKD. While the all-cause mortality rate in patients with ESKD tended to increase, the difference was not statistically significant. Additionally, no statistically significant differences in glucocorticoids and immunosuppressive drugs administered after AAV-GN between patients with ESKD and those without were observed (Table 1).

### Cross-sectional comparison of histologic features at AAV-GN diagnosis between patients with ESKD and those without ESKD

Among 113 patients, 23, 28, 34 and 28 patients had focal, crescentic, mixed and sclerotic classes based on histopathological classification, respectively^12^. The frequency of focal class was significantly higher in the patients without ESKD, whereas that of the sclerotic class was significantly higher in the patients with ESKD. Patients with ESKD showed a higher proportion of global glomerular sclerosis than those without ESKD (35.7% vs. 22.9%, P = 0.010). However, there were no significant differences in crescent formation between the two groups. In terms of specific histologic lesion scores, compared to patients without ESKD, those with ESKD significantly exhibited higher frequencies of high scores in three items such as interstitial fibrosis, tubular atrophy, and global glomerulitis (Table 2).

**Table 2:** Comparison of histopathologic features between patients with ESKD and those without ESKD.

Variables	Patients without ESKD (N = 63)	Patients with ESKD (N = 50)	P value
Histopathologic classification (N, (%))			
Focal	18 (28.6)	5 (10.0)	0.015
Crescentic	13 (20.6)	15 (30.0)	0.252
Mixed	22 (34.9)	12 (24.0)	0.209
Sclerotic	10 (15.9)	18 (36.0)	0.014
Glomerular sclerosis^†^			
Global	22.9%	35.7%	0.010
Segmental	6.5%	11.1%	0.044
Crescent formation^†^			
Cellular	19.6%	21.5%	0.293
Fibrous	1.8%	4.6%	0.080
Fibrocellular	11.1%	14.1%	0.351
Total (cellular + fibrous + fibrocellular)	32.5%	40.2%	0.088

Histologic lesion scores (N, (%))	Patients without ESKD (N = 62)	Patients with ESKD (N = 50)	P value
Acute tubular injury			
Absent	26 (41.9)	28 (56.0)	0.139
Present	36 (58.1)	22 (44.0)	
Medullary angiitis			
Absent	25 (40.3)	19 (38.0)	0.802
Present	37 (59.7)	31 (62.0)	
Arterial intimal fibrosis			
Absent	43 (69.4)	29 (58.0)	0.212
Present	19 (30.6)	21 (42.0)	
Arterial medial sclerosis			
Absent	59 (95.2)	42 (84.0)	0.060
Present	3 (4.8)	8 (16.0)	
Interstitial inflammation			
Low (0–1)	35 (56.5)	27 (54.0)	0.795
High (2–3)	27 (43.5)	23 (46.0)	
Tubulitis			
Low (0–1)	37 (59.7)	30 (60.0)	0.972
High (2–3)	25 (40.3)	20 (40.0)	
Vasculitis			
Low (0–1)	51 (82.3)	46 (92.0)	0.132
High (2–3)	11 (17.7)	4 (8.0)	
Glomerulitis			
Low (0–1)	57 (91.9)	46 (92.0)	0.990
High (2–3)	5 (8.1)	4 (8.0)	
Peritubular capillaritis			
Low (0–1)	36 (58.1)	28 (56.0)	0.826
High (2–3)	26 (41.9)	22 (44.0)	
Total inflammation			
Low (0–1)	28 (45.2)	21 (42.0)	0.737
High (2–3)	34 (54.8)	29 (58.0)	
Inflammation in IFTA			
Low (0–1)	25 (40.3)	20 (40.0)	0.972
High (2–3)	37 (59.7)	30 (60.0)	
GBM double contour			
Low (0)	60 (96.8)	49 (98.0)	1.000
High (1–3)	2 (3.2)	1 (2.0)	
Arteriolar hyalinosis			
Low (0–1)	62 (100)	49 (98.0)	0.446
High (2–3)	0 (0)	1 (2.0)	
Arteriosclerosis			
Low (0–1)	52 (83.9)	38 (76.0)	0.297
High (2–3)	10 (16.1)	12 (24.0)	
Interstitial fibrosis			
Low (0–1)	50 (80.6)	27 (54.0)	0.002
High (2–3)	12 (19.4)	23 (46.0)	
Tubular atrophy			
Low (0–1)	44 (71.0)	21 (42.0)	0.002
High (2–3)	18 (29.0)	29 (58.0)	
Mesangial hypercellularity			
Low (0–1)	59 (95.2)	49 (98.0)	0.627
High (2–3)	3 (4.8)	1 (2.0)	
Global glomerulitis			
Low (0)	60 (96.8)	42 (84.0)	0.041
High (1–3)	2 (3.2)	8 (16.0)	
Glom. fibrinoid necrosis			
Low (0)	32 (51.6)	32 (64.0)	0.188
High (1–3)	30 (48.4)	18 (36.0)	

Values are expressed as a median (interquartile range, IQR) or N (%).

*AAV* ANCA-associated vasculitis, *ANCA* antineutrophil cytoplasmic antibody, *ESKD* end-stage kidney disease, *IFTA* interstitial fibrosis and tubular atrophy, *GBM* glomerular basement membrane.

^†^Mean percentage of each group. For each patient, the percentage was calculated by dividing the number of lesional glomeruli by the total number of glomeruli.

### Comparison of the cumulative ESKD-free survival rates among the four classes based on the histopathological classification

**Figure 1:**
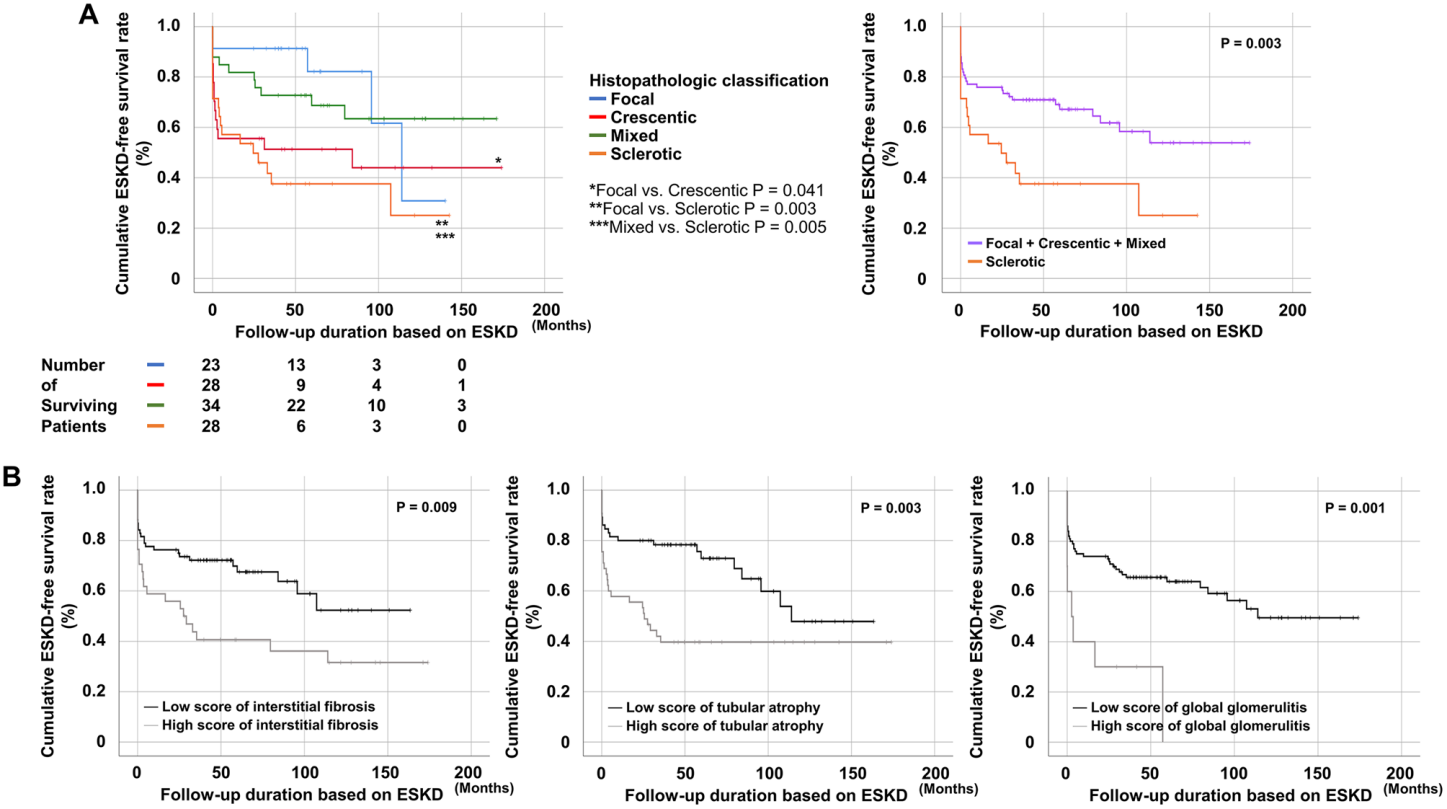
Comparison of the cumulative ESKD-free survival rates. (**A**) The sclerotic class exhibited the lowest ESKD-free survival rate among the four histopathological classes in patients with AAV-GN. (**B**) Patients with high scores of interstitial fibrosis, tubular atrophy and endocapillary hypercellularity showed significantly lower cumulative ESKD-free survival rates compared to those with low scores. *ESKD* end-stage kidneye disease, *AAV-GN* antineutrophil cytoplasmic antibody-associated vasculitis-glomerulonephritis.

The highest cumulative ESKD-free survival rate was observed for the focal class, followed by the mixed, crescentic and sclerotic classes. When the cumulative ESKD-survival rates of the two classes were compared, there were two clinically significant comparisons. Patients in the focal class exhibited a significantly higher cumulative ESKD-free survival rate than those in the sclerotic (P = 0.003) classes. In addition, the cumulative ESKD-free survival rate of patients in the mixed class was significantly higher than that of patients in the sclerotic class (P = 0.005). Moreover, when compared between the sclerotic class and the remaining three classes (focal + crescentic + mixed), patients in the sclerotic class exhibited a significantly reduced cumulative ESKD-survival rate compared to those in the remaining three classes (P = 0.003) (Fig. 1A).

### Comparison of the cumulative ESKD-free survival rates according to each item of histologic lesion scores

Among the histologic lesion scores, cumulative ESKD-free survival rates differed significantly between patients with high scores and those with low scores on interstitial fibrosis (P = 0.009), tubular atrophy (P = 0.003), and global glomerulitis (P = 0.001). Patients with high scores on all three items showed significantly lower cumulative ESKD-free survival rates compared to the rate in patients with low scores (Fig. 1B).

### Cox hazards model analysis among histopathological features

In the univariable Cox hazards model analysis, high scores for interstitial fibrosis (HR 2.074), tubular atrophy (HR 2.259), and global glomerulitis (HR 3.311) were significantly associated with the progression to ESKD in patients with AAV-GN. In the multivariable analysis, only high global glomerulitis scores were independently and significantly associated with the progression to ESKD (HR 3.164, 95% confidence interval (CI) 1.440, 6.949, P = 0.004) (Table 3).

**Table 3:** Cox hazards model analysis of variables regarding histologic lesion scores for ESKD during follow-up in patients AAV-GN.

Variables	Univariable			Multivariable		
	HR	95% CI	P value	HR	95% CI	P value
Acute tubular injury	0.688	0.387, 1.223	0.202			
Medullary angiitis	1.037	0.577, 1.863	0.903			
Arterial intimal fibrosis	1.600	0.903, 2.834	0.108			
Arterial medial sclerosis	1.933	0.903, 4.135	0.089			
Interstitial inflammation	1.216	0.688, 2.152	0.501			
Tubulitis	1.070	0.598, 1.912	0.820			
Vasculitis	0.562	0.201, 1.570	0.272			
Glomerulitis	1.061	0.381, 2.955	0.910			
Peritubular capillaritis	1.137	0.642, 2.013	0.661			
Total inflammation	1.215	0.684, 2.161	0.506			
Inflammation in IFTA	1.026	0.578, 1.822	0.930			
GBM double contour	0.726	0.100, 5.272	0.752			
Arteriolar hyalinosis	5.576	0.746, 41.663	0.094			
Arteriosclerosis	1.501	0.780, 2.889	0.224			
Interstitial fibrosis	2.074	1.171, 3.674	0.012	1.335	0.638, 2.792	0.443
Tubular atrophy	2.259	1.274, 4.066	0.005	1.878	0.898, 3.927	0.094
Mesangial hypercellularity	0.506	0.070, 3.671	0.501			
Global glomerulitis	3.311	1.520, 7.209	0.003	3.164	1.440, 6.949	0.004
Glomerular fibrinoid necrosis	0.726	0.402, 1.312	0.289			

*ESKD* end-stage kidney disease, *AAV* ANCA-associated vasculitis, *ANCA* antineutrophil cytoplasmic antibody, *GN* glomerulonephritis, *UPIGN* unclassifiable pauci-immune glomerulonephritis, *IFTA* interstitial fibrosis and tubular atrophy.

### Cox hazards model analysis among clinical and histopathological features

The clinical variables with statistical significance in the cross-sectional comparison analysis or known clinical risk factors for ESKD were also included in the Cox analysis together with the variables related to the histologic lesion scores. In the univariable Cox hazards model analysis, BUN (HR 1.016), serum creatinine (HR 1.406), uric acid (HR 1.195), urine protein-to-creatinine ratio (HR 1.141), and high scores for interstitial fibrosis (HR 2.074), tubular atrophy (HR 2.259), and global glomerulitis (HR 3.311) were significantly associated with the progression to ESKD in patients with AAV-GN. Since eGFR was calculated using four parameters (sex, age, serum creatinine and race), which were analysed as clinical variables, eGFR was not included in the analysis in this study. In the multivariable analysis, BUN (HR 0.964, 95% CI 0.940, 0.988), serum creatinine (HR 1.830, 95% CI 1.454, 2.302) and high global glomerulitis score (HR 3.325, 95% CI 1.373, 8.050) were independently and significantly associated with the progression to ESKD (Table 4).

**Table 4:** Cox hazards model analysis of variables regarding clinical features and histologic lesion scores with significance in univariable analysis for the progression to ESKD during follow-up in patients AAV-GN.

Variables	Univariable			Multivariable		
	HR	95% CI	P value	HR	95% CI	P value
Age (years)	1.000	0.982, 1.018	0.998			
Male gender (N, (%))	1.384	0.769, 2.493	0.278			
MPO-ANCA (or P-ANCA) positivity	0.732	0.396, 1.350	0.318			
PR3-ANCA (or C-ANCA) positivity	1.742	0.740, 4.103	0.204			
Haemoglobin (g/dL)	0.855	0.700, 1.045	0.126			
BUN (mg/dL)	1.016	1.006, 1.026	0.002	0.964	0.940, 0.988	0.004
Serum creatinine (mg/dL)	1.406	1.238, 1.559	< 0.001	1.830	1.454, 2.302	< 0.001
Uric acid	1.195	1.039, 1.374	0.012	1.186	0.936, 1.503	0.157
ESR (mm/h)	0.994	0.985, 1.002	0.157			
CRP (mg/L)	0.994	0.986, 1.001	0.102			
Urine protein-to-creatinine ratio	1.141	1.052, 1.237	0.002	1.080	0.974, 1.197	0.144
Haematuria	1.118	0.556, 2.245	0.755			
C3 (mg/dL)	1.001	0.998, 1.004	0.496			
C4 (mg/dL)	1.000	0.974, 1.027	0.996			
Interstitial fibrosis	2.074	1.171, 3.674	0.012	0.815	0.385, 1.727	0.594
Tubular atrophy	2.259	1.274, 4.066	0.005	1.804	0.853, 3.812	0.122
Global glomerulitis	3.311	1.520, 7.209	0.003	3.325	1.373, 8.050	0.008

eGFR (EPI) is calculated using 4 parameters, sex, age, serum creatinine and race (all Korean patients (non-black)). Therefore, eGFR was not included in this analysis because age, sex and serum creatinine were analysed in this analysis.

*ESKD* end-stage kidney disease, *AAV* ANCA-associated vasculitis, *ANCA* antineutrophil cytoplasmic antibody, *GN* glomerulonephritis, *MPO* myeloperoxidase, *P* perinuclear, *PR3* proteinase 3, *C* cytoplasmic, *BUN* blood urea nitrogen, *ESR* erythrocyte sedimentation rate, *CRP* C-reactive protein, *C3* complement 3, *C4* complement 4.

## Discussion

This study investigated which histopathological features could significantly and independently predict the progression to ESKD in patients with AAV-GN and obtained several interesting findings. First, in terms of the rate of ESKD occurrence, 44.2% of patients with AAV-GN confirmed by kidney biopsy showed progression to ESKD during follow-up. Second, in terms of clinical features, the cross-sectional comparison analysis revealed significant differences in several kidney-, inflammation-, and AAV-pathogenesis-related variables between patients with ESKD and those without ESKD. Third, in terms of histopathological classification and histologic lesion scores, the sclerotic class exhibited the worst renal prognosis among the four histopathological classes. Among histopathological features, in the univariable Cox proportional analysis, high interstitial fibrosis, tubular atrophy and global glomerulitis scores were significantly associated with ESKD progression, whereas in the multivariable analysis, only a high global glomerulitis score was an independent predictor of ESKD progression. Finally, in terms of clinical and histologic lesion scores, a high global glomerulitis score could also predict ESKD progression in addition to serum BUN and creatinine levels at AAV-GN diagnosis.

Because the number of patients with EGPA-GN and GPA-GN was small, it might be difficult to analyse them by dividing them into 5 groups. We excluded 5 and 8 patients with EGPA-GN, and GPA-GN, respectively, and performed the univariable and multivariable Cox analyses in the remaining 100 patients again. In the univariable Cox analysis of variables regarding histologic lesion scores for ESKD during follow-up in 100 patients with AAV-GN excluding patients with EGPA-GN, and GPA-GN, interstitial fibrosis was not significantly associated with ESKD. Instead, arterial intimal fibrosis was significantly associated with ESKD. In the multivariable Cox analysis, in addition to global glomerulitis (HR 3.253, 95% CI 1.410, 7.505), tubular atrophy (HR 2.123, 95% CI 1.133, 3.977) was also independently associated with ESKD (Supplementary Table S1). Also, in the multivariable Cox analysis, of variables regarding clinical features and histologic lesion scores with significance in univariable analysis for the progression to ESKD during follow-up in 100 patients with AAV-GN excluding patients with EGPA-GN, and GPA-GN, in addition to serum creatinine (HR 1.794, 95% CI 1.407, 2.287), only global glomerulitis was independently associated with ESKD (HR 3.157, 1.234, 8.076) (Supplementary Table S2). However, we hope to include patients with EGPA-GN and GPA-GN patients in this study for three reasons: first, in both analyses including or excluding patients with EGPA-GN and GPA-GN, only global glomerulitis was independently associated with ESKD; second, in real clinical practice, it may be sometimes difficult to clearly distinguish AAV subtypes in one patient; and third, there are also occasional cases in which the AAV subtype changes in one patient during the follow-up period owing to the change and update of the classification criteria for AAV.

Although the difference between the focal and mixed classes has been questioned^20–22^, the Kaplan Meier curve for renal survival according to histopathological classification in the present study demonstrated a different pattern from those described previously^12,13^. The survival curve of the focal class crossed over the mixed and crescentic classes, finally reaching worse survival than those of the other two classes. In other studies, the focal class retained the best survival among the four classes during follow-up^12,13^. First, this may occur due to the heterogeneous nature of the focal class, which may include glomeruli with an underlying pathology that is not yet fully observable by light microscopy. Second, the classification originally suggested a minimum requirement of 10 glomeruli in kidney biopsy samples. However, the present study did not exclude kidney biopsy samples with < 10 glomeruli, thereby allowing little chance of including glomeruli with true lesions, such as crescents or glomerulosclerosis, and erroneously classifying them into the focal class. For example, if the total number of glomeruli obtained from a biopsy is 9, and 4 of them are normal, it would not be classified as focal class. However, if the total number of glomeruli obtained is 8, and 4 of them are normal, it would be classified as focal class. In other words, the classification changes with the number of normal glomeruli changes by one where the total number of glomeruli is less than 10, but it is doubtful whether the change is meaningful enough to represent the unsampled kidney. The histopathological classification itself also has limitations as it does not include tubulointerstitial or vascular lesions. Moreover, the therapeutic effects on renal survival have not been considered^12^.

The results of the multivariable Cox hazard model analysis revealed that BUN, serum creatinine, and global glomerulitis were independently associated with the progression to ESKD in patients with AAV-GN. Since both BUN and serum creatinine are well-known clinical risk factors for ESKD occurrence along with eGFR, it was not unexpected that they were independent predictors of ESKD. Moreover, this finding also supported the validity of the analysis in the present study. Among the variables related to histopathological lesion scores, only global glomerulitis was a significant and independent predictor for the progression to ESKD in patients with AAV-GN^23–26^. Consistent with previous findings, the results of the univariable Cox analysis in the present study also demonstrated the predictive power of interstitial fibrosis and tubular atrophy for the progression to ESKD in patients with AAV-GN. Notably, global glomerulitis was more powerful than interstitial fibrosis and tubular atrophy in predicting ESKD. Glomerulitis or endocapillary hypercellularity (though not all endocapillary hypercellularity corresponds to glomerulitis because endocapillary hypercellularity includes mesangial or endothelial proliferation) has been described as a significant histologic parameter in some kidney diseases. Endocapillary hypercellularity was predictive of poorer renal survival in patients with IgA nephropathy not treated with immunosuppressants^27^. It was also associated with better renal outcomes when treated with immunosuppressants in patients with IgA nephropathy^28,29^ or lupus nephritis^30^, reflecting the reversible and active nature of the lesion. Endocapillary hypercellularity may result from immune-complex deposition on the capillary wall along with an activated alternative complement pathway. An influx of leukocytes releases cytokines and proteases, leading to endothelial injury and capillary damage due to proteinuria and haematuria^31^. Although glomerulitis itself did not reveal significant association with ESKD in the present study, narrowing the variable into global glomerulitis, a more severe form of glomerulitis, revealed a significance in predicting ESKD in patients with AAV-GN. Moreover, receiving treatment was not included in analysis as a variable, future studies may find more information on the association of global glomerulitis with ESKD after treatment.

The frequency of the sclerotic class based on histopathological classification and the proportion of global glomerular sclerosis at the time of kidney biopsy were significantly higher in patients with ESKD than in those without ESKD (Table 2). Therefore, these factors might contribute to the assessment of the progression to ESKD in AAV-GN. When they were included in the univariable Cox hazard model analysis along with significant variables related to clinical features and histologic lesion scores in univariable analysis, the sclerotic class (HR 2.299) and global glomerular sclerosis (HR 1.016) were significantly associated with the progression to ESKD. However, they were not independent predictors of ESKD in the multivariable Cox analysis. BUN (HR 0.961, 95% CI 0.937, 0.985), serum creatinine (HR 1.911, 95% CI 1.512, 2.416) and global glomerulitis (HR 3.781, 95% CI 1.514, 9.445) were also significantly and independently associated with the progression to ESKD (Supplementary Table S3). Thus, close follow-up and prompt intervention in patients with low BUN, high creatinine, and global glomerulitis in renal biopsy might prevent the progression to ESKD.

On the other hand, it is already well known that the sclerosing type has a poor prognosis. Therefore, we excluded 28 patients with sclerotic type and performed the Cox analyses again. In the univariable Cox analysis, only global glomerulitis was significantly associated with ESKD, and thus, the multivariable Cox analysis could not be performed (Supplementary Table S4). Therefore, we conclude that similar to the results analysed in all 113 patients, global glomerulitis is a major predictor of ESKD occurrence during follow-up, regardless of the inclusion of patients with sclerotic type.

In addition, we investigated the clinical significance of normal glomeruli at the time of the kidney biopsy in the progression to ESKD during follow-up in patients with AAV-GN by performing the univariable and multivariable Cox analyses using the % normal glomeruli, % crescents and % sclerotic glomeruli. In the univariable analysis, among the three variables, global glomerulosclerosis (%) (HR 1.016) and normal (%) (HR 0.972) were significantly associated with ESKD, whereas, in the multivariable analysis, only normal (%) was independently associated with ESKD during follow-up in patients with AAV-GN (HR 0.975, 95% CI 0.958, 0.992) (Supplementary Table S5). Therefore, it is concluded that a finding of normal glomeruli at the time of the kidney biopsy may play a protective role in the progression to ESKD during follow-up in patients with AAV-GN.

Among studies that have investigated the association between histopathological signs and ESKD^32^, some studies have utilized parts of the Banff scoring system to assess the risk of ESKD^33–35^. However, not only have we adopted the entire Banff scoring system, but also incorporated new item such as global glomerulitis. Thus, the strength of this study is that this is the first to extensively evaluate all three components (glomerular, tubulointerstitial, and vascular lesions) that constitute renal pathology, and evaluate the risk of ESKD. In addition, this study first discovered that a high global glomerulitis was an independent predictor of ESKD in patients with AAV-GN. This study has several limitations. The number of patients was not sufficiently large to represent Korean patients with AAV-GN and the statistical power of the retrospective study design was not as high as that of a prospective study. The follow-up period was not long enough to confirm future differentiation of the AAV-GN subtype in patients with UPIGN, and the interobserver variation among the pathologists was not evaluated. Additionally, another limitation is that the "10 events per variable" rule, which is a common heuristic to balance model complexity and statistical power, was not obeyed in the Cox analyses. However, given that it is not a strict rule and can vary based on the study context, and we considered factors like variable importance and interactions, it is believed that this limitation might be tolerated. Future prospective studies with larger numbers of patients and serial collections of clinical data will overcome these limitations and provide more reliable and clearer information on the histopathological predictors of the progression to ESKD in patients with AAV-GN.

In conclusion, this study demonstrated the worst renal prognosis for the sclerotic class among the four histopathological classes. High histological lesion scores for interstitial fibrosis, tubular atrophy, and global glomerulitis were significantly associated with the progression to ESKD in Korean patients with AAV-GN. Finally, this study is the first to show that global glomerulitis independently predicted the progression to ESKD in Korean patients with AAV-GN. Therefore, more careful monitoring and shorter follow-ups are needed in patients with AAV-GN with high global glomerulitis scores in the biopsy findings.

### Supplementary Information

Supplementary Information.

## Data Availability

Data and material can be requested from Sang-Won Lee (sangwonlee@yuhs.ac) on reasonable request.

## References

[1] Jennette JC (2013). 2012 revised international Chapel Hill consensus conference nomenclature of vasculitides. Arthritis Rheum.

[2] Watts R (2007). Development and validation of a consensus methodology for the classification of the ANCA-associated vasculitides and polyarteritis nodosa for epidemiological studies. Ann. Rheum. Dis..

[3] Jennette, J. C. *et al.* Nomenclature of systemic vasculitides. Proposal of an international consensus conference. *Arthritis Rheum* **37,** 187–192 (1994). 10.1002/art.1780370206 8129773

[4] Cornec, D., Cornec-Le Gall, E., Fervenza, F. C. & Specks, U. ANCA-associated vasculitis—clinical utility of using ANCA specificity to classify patients. *Nat. Rev. Rheumatol.* **12,** 570–579 (2016). 10.1038/nrrheum.2016.123 27464484

[5] Hakroush S, Tampe D, Strobel P, Korsten P, Tampe B (2021). Comparative histological subtyping of immune cell infiltrates in MPO-ANCA and PR3-ANCA glomerulonephritis. Front. Immunol..

[6] Millet A, Pederzoli-Ribeil M, Guillevin L, Witko-Sarsat V, Mouthon L (2014). Republished: Antineutrophil cytoplasmic antibody-associated vasculitides: Is it time to split up the group?. Postgrad. Med. J..

[7] Kim MK (2020). Multivariable index for assessing the activity and predicting all-cause mortality in antineutrophil cytoplasmic antibody-associated vasculitis. J. Clin. Lab. Anal..

[8] Park, P. G. *et al.* Metabolic syndrome severity score, comparable to serum creatinine, could predict the occurrence of end-stage kidney disease in patients with antineutrophil cytoplasmic antibody-associated vasculitis. *J. Clin. Med.* **10** (2021). 10.3390/jcm10245744 PMC8708376 34945043

[9] Wang, R. *et al.* Clinicopathological characteristics and influencing factors of renal vascular lesions in anti-neutrophil cytoplasmic autoantibody-related renal vasculitis. *Front. Med. (Lausanne)* **8,** 710386 (2021). 10.3389/fmed.2021.710386 PMC8505665 34650993

[10] Solbakken V, Fismen AS, Bostad L, Bjorneklett R (2018). Impact of proteinase 3 versus myeloperoxidase positivity on risk of end-stage renal disease in ANCA-associated glomerulonephritis stratified by histological classification: A population-based cohort study. Dis. Markers.

[11] Brix SR (2018). Development and validation of a renal risk score in ANCA-associated glomerulonephritis. Kidney Int..

[12] Berden AE (2010). Histopathologic classification of ANCA-associated glomerulonephritis. J. Am. Soc. Nephrol..

[13] Lim JH (2021). Histopathologic and clinicopathologic classifications of antineutrophil cytoplasmic antibody-associated glomerulonephritis: A validation study in a Korean cohort. Kidney Res. Clin. Pract..

[14] Sato N (2017). Renal-limited vasculitis with elevated levels of multiple antibodies. CEN Case Rep..

[15] Naidu GS (2014). Histopathological classification of pauci-immune glomerulonephritis and its impact on outcome. Rheumatol. Int..

[16] Agarwal R (2016). Defining end-stage renal disease in clinical trials: A framework for adjudication. Nephrol. Dial Transplant.

[17] Bajema IM (2018). Revision of the International Society of Nephrology/Renal Pathology Society classification for lupus nephritis: clarification of definitions, and modified National Institutes of Health activity and chronicity indices. Kidney Int..

[18] Roufosse C (2018). A 2018 reference guide to the Banff classification of renal allograft pathology. Transplantation.

[19] Working Group of the International Ig, A. N. N. *et al.* The Oxford classification of IgA nephropathy: Pathology definitions, correlations, and reproducibility. *Kidney Int.* **76,** 546–556 (2009). 10.1038/ki.2009.168 19571790

[20] Bjorneklett R, Sriskandarajah S, Bostad L (2016). prognostic value of histologic classification of ANCA-associated glomerulonephritis. Clin. J. Am. Soc. Nephrol..

[21] Cordova-Sanchez BM (2016). Clinical presentation and outcome prediction of clinical, serological, and histopathological classification schemes in ANCA-associated vasculitis with renal involvement. Clin. Rheumatol..

[22] Moroni, G. *et al.* Predictors of renal survival in ANCA-associated vasculitis. Validation of a histopatological classification schema and review of the literature. *Clin. Exp. Rheumatol.* **33,** S56–S63 (2015). 26016751

[23] Bajema IM (1999). Kidney biopsy as a predictor for renal outcome in ANCA-associated necrotizing glomerulonephritis. Kidney Int..

[24] Tanna A (2015). Long-term outcome of anti-neutrophil cytoplasm antibody-associated glomerulonephritis: Evaluation of the international histological classification and other prognostic factors. Nephrol. Dial Transplant.

[25] de Lind van Wijngaarden, R. A. *et al.* Clinical and histologic determinants of renal outcome in ANCA-associated vasculitis: A prospective analysis of 100 patients with severe renal involvement. *J. Am. Soc. Nephrol.* **17,** 2264–2274 (2006). 10.1681/ASN.2005080870 16825335

[26] Hauer HA (2002). Determinants of outcome in ANCA-associated glomerulonephritis: a prospective clinico-histopathological analysis of 96 patients. Kidney Int..

[27] Chakera A (2016). Prognostic value of endocapillary hypercellularity in IgA nephropathy patients with no immunosuppression. J. Nephrol..

[28] Trimarchi H (2017). Oxford classification of IgA nephropathy 2016: An update from the IgA nephropathy classification working group. Kidney Int..

[29] Shen XH (2015). Reversal of active glomerular lesions after immunosuppressive therapy in patients with IgA nephropathy: A repeat-biopsy based observation. J. Nephrol..

[30] Obrisca B (2018). Histological predictors of renal outcome in lupus nephritis: the importance of tubulointerstitial lesions and scoring of glomerular lesions. Lupus.

[31] Sethi S, Rajkumar SV (2013). Monoclonal gammopathy-associated proliferative glomerulonephritis. Mayo Clin. Proc..

[32] Boud'hors C (2022). Histopathological prognostic factors in ANCA-associated glomerulonephritis. Autoimmun. Rev..

[33] Bitton L (2020). Tubulointerstitial damage and interstitial immune cell phenotypes are useful predictors for renal survival and relapse in antineutrophil cytoplasmic antibody-associated vasculitis. J. Nephrol..

[34] Quintana LF (2014). ANCA serotype and histopathological classification for the prediction of renal outcome in ANCA-associated glomerulonephritis. Nephrol. Dial Transplant.

[35] Nohr E, Girard L, James M, Benediktsson H (2014). Validation of a histopathologic classification scheme for antineutrophil cytoplasmic antibody-associated glomerulonephritis. Hum. Pathol..

