# Supplementary material for: Renal histopathological predictors of end-stage kidney disease in ANCA-associated vasculitis with glomerulonephritis: a single-centre study in Korea
Source: Sci Rep. 2023 Sep 8;13:14850. doi: 10.1038/s41598-023-41811-0 (PMC10491748; doi:10.1038/s41598-023-41811-0)
Supplement: Supplementary file 1 — Supplementary Information. [file 41598_2023_41811_MOESM1_ESM.pdf]

Title: Renal histopathological predictors of end-stage kidney disease in ANCA-associated vasculitis with glomerulonephritis: a single-centre study in Korea

Authors: Sung-Eun Choi [1*], Soo Bin Lee[2*], Jung Yoon Pyo[3], Sung Soo Ahn[3], Jason Jungsik Song[3,4], Yong-Beom Park[3,4], Beom Jin Lim [5**], and Sang-Won Lee[3,4**]

Supplementary Methods

The components of the Banff scoring system used in this study were interstitial inflammation (i), tubulitis (t), vasculitis (v), glomerulitis (g), peritubular capillaritis (ptc), total inflammation (i), inflammation in the area of interstitial fibrosis and tubular atrophy (i-IFTA), glomerular basement membrane double contour (cg), arteriolar hyalinosis (ah), arteriosclerosis (cv), interstitial fibrosis (ci), and tubular atrophy (ct). Mesangial hypercellularity was scored according to the definitions from the Oxford study (18). In addition to histological lesions identical to the Banff scoring system, which has three grades, mesangial hypercellularity and global glomerulitis were subclassified into three grades; 0,1,2, and 3. That is, normal mesangial hypercellularity was scored as 0, mild as 1, moderate as 2, and severe as 3.

Supplementary Table 1 Cox hazards model analysis of variables regarding histologic lesion scores for ESKD during follow-up in 100 patients AAV-GN excluding patients with EGPA-GN, and GPA-GN

**Variables**	**Univariable**			**Multivariable**		
	**HR**	**95% CI**	**P value**	**HR**	**95% CI**	**P value**
Acute tubular injury	0.598	0.323, 1.109	0.103			
Medullary angiitis	1.054	0.558, 1.990	0.871			
Arterial intimal fibrosis	1.870	1.014, 3.449	0.045	1.666	0.894, 3.106	0.108
Arterial medial sclerosis	2.161	0.957, 4.877	0.064			
Interstitial inflammation	1.270	0.692, 2.333	0.441			
Tubulitis	1.051	0.562, 1.963	0.877			
Vasculitis	0.495	0.153, 1.608	0.242			
Glomerulitis	0.976	0.301, 3.163	0.968			
Peritubular capillaritis	1.109	0.601, 2.046	0.740			
Total inflammation	1.205	0.650, 2.234	0.554			
Inflammation in IFTA	1.066	0.575, 1.976	0.840			
GBM double contour	1.709	0.234, 12.486	0.598			
Arteriolar hyalinosis	5.879	0.780, 44.342	0.086			
Arteriosclerosis	1.509	0.740, 3.077	0.258			
Interstitial fibrosis	1.783	0.957, 3.321	0.069			
Tubular atrophy	2.279	1.231, 4.218	0.009	2.123	1.133, 3.977	0.019
Mesangial hypercellularity	0.505	0.069, 3.677	0.500			
Global glomerulitis	3.238	1.412, 7.422	0.006	3.253	1.410, 7.505	0.006
Glomerular fibrinoid necrosis	0.653	0.340, 1.258	0.203			

ESKD: end-stage kidney disease; AAV: ANCA-associated vasculitis; ANCA: antineutrophil cytoplasmic antibody; GN: glomerulonephritis; UPIGN: unclassifiable pauci-immune glomerulonephritis; IFTA: interstitial fibrosis and tubular atrophy.

Supplementary Table 2 Cox hazards model analysis of variables regarding clinical features and histologic lesion scores with significance in univariable analysis for the progression to ESKD during follow-up in 100 patients AAV-GN* excluding patients with EGPA-GN, and GPA-GN

**Variables**	**Univariable**			**Multivariable**		
	**HR**	**95% CI**	**P value**	**HR**	**95% CI**	**P value**
Age (years)	1.002	0.983, 1.021	0.820			
Male gender (N, (%))	1.413	0.755, 2.646	0.280			
MPO-ANCA (or P-ANCA) positivity	0.771	0.393, 1.514	0.450			
PR3-ANCA (or C-ANCA) positivity	2.167	0.667, 7.038	0.198			
Haemoglobin (g/dL)	0.854	0.694, 1.049	0.132			
BUN (mg/dL)	1.019	1.008, 1.029	<0.001	0.975	0.950, 1.001	0.062
Serum creatinine (mg/dL)	1.430	1.281, 1.597	<0.001	1.794	1.407, 2.287	<0.001
Uric acid	1.213	1.043, 1.411	0.012	1.118	0.873, 1.431	0.377
ESR (mm/h)	0.996	0.986, 1.005	0.337			
CRP (mg/L)	0.994	0.986, 1.003	0.171			
Urine protein-to-creatinine ratio	1.127	1.030, 1.232	0.009	1.066	0.953, 1.192	0.261
Haematuria	1.118	0.517, 2.419	0.777			
C3 (mg/dL)	1.001	0.999, 1.004	0.259			
C4 (mg/dL)	1.005	0.976, 1.034	0.757			
Arterial intimal fibrosis	1.870	1.014, 3.449	0.045	1.815	0.885, 3.720	0.104
Tubular atrophy	2.279	1.231, 4.218	0.009	1.626	0.810, 3.265	0.172
Global glomerulitis	3.238	1.412, 7.422	0.006	3.157	1.234, 8.076	0.016

ESKD: end-stage kidney disease; AAV: ANCA-associated vasculitis; ANCA: antineutrophil cytoplasmic antibody; GN: glomerulonephritis; MPO: myeloperoxidase; P: perinuclear; PR3: proteinase 3; C: cytoplasmic; BUN: blood urea nitrogen; ESR: erythrocyte sedimentation rate; CRP: C-reactive protein; C3: complement 3; C4: complement 4.

*eGFR (EPI) is calculated using 4 parameters, sex, age, serum creatinine and race (all Korean patients (non-black)). Therefore, eGFR was not included in this analysis because age, sex and serum creatinine were analysed in this analysis.

Supplementary Table 3 Cox hazards model analysis of variables regarding histopathologic features, clinical features, and histologic lesion scores with significance in univariable analysis for the progression to ESKD during follow-up in patients AAV-GN*

Variables	Univariable			Multivariable		
	**HR**	**95% CI**	**P value**	**HR**	**95% CI**	**P value**
BUN (mg/dL)	1.016	1.006, 1.026	0.002	0.961	0.937, 0.985	0.002
Serum creatinine (mg/dL)	1.406	1.238, 1.559	<0.001	1.911	1.512, 2.416	<0.001
Uric acid	1.195	1.039, 1.374	0.012	1.169	0.927, 1.475	0.187
Urine protein-to-creatinine ratio	1.141	1.052, 1.237	0.002	1.099	0.992, 1.217	0.072
Interstitial fibrosis	2.074	1.171, 3.674	0.012	0.695	0.311, 1.553	0.375
Tubular atrophy	2.259	1.274, 4.066	0.005	1.395	0.622, 3.132	0.419
Global glomerulitis	3.311	1.520, 7.209	0.003	3.781	1.514, 9.445	0.004
Sclerotic class based on the histopathologic classification	2.299	1.274, 4.149	0.006	0.770	0.221, 2.684	0.681
Global glomerular sclerosis	1.016	1.005, 1.028	0.004	1.018	0.993, 1.043	0.166

ESKD: end-stage kidney disease; AAV: ANCA-associated vasculitis; ANCA: antineutrophil cytoplasmic antibody; GN: glomerulonephritis; BUN: blood urea nitrogen.
*eGFR (EPI) is calculated using 4 parameters, sex, age, serum creatinine and race (all Korean patients (non-black)). Therefore, eGFR was not included in this analysis because age, sex and serum creatinine were analysed in this analysis.

Supplementary Table 4 Cox hazards model analysis of variables regarding histologic lesion scores for ESKD during follow-up in 85 patients AAV-GN excluding 28 patients with sclerotic type

Variables	Univariable		
	**HR**	**95% CI**	**P value**
Acute tubular injury	0.732	0.355, 1.508	0.398
Medullary angiitis	1.514	0.673, 3.403	0.316
Arterial intimal fibrosis	1.534	0.737, 3.193	0.252
Arterial medial sclerosis	1.877	0.653, 5.397	0.242
Interstitial inflammation	1.101	0.532, 2.276	0.796
Tubulitis	1.249	0.603, 2.585	0.549
Vasculitis	0.563	0.170, 1.865	0.347
Glomerulitis	1.377	0.480, 3.951	0.552
Peritubular capillaritis	1.251	0.609, 2.572	0.542
Total inflammation	1.098	0.535, 2.257	0.798
Inflammation in IFTA	0.874	0.424, 1.803	0.716
GBM double contour	0.047	0.000, 421.818	0.511
Arteriolar hyalinosis	7.124	0.920, 55.194	0.060
Arteriosclerosis	1.864	0.797, 4.358	0.151
Interstitial fibrosis	1.711	0.796, 3.679	0.169
Tubular atrophy	2.019	0.978, 4.166	0.057
Mesangial hypercellularity	0.047	0.000, 421.818	0.511
Global glomerulitis	3.713	1.383, 9.967	0.009
Glomerular fibrinoid necrosis	0.974	0.476, 1.995	0.944

ESKD: end-stage kidney disease; AAV: ANCA-associated vasculitis; ANCA: antineutrophil cytoplasmic antibody; GN: glomerulonephritis; UPIGN: unclassifiable pauci-immune glomerulonephritis; IFTA: interstitial fibrosis and tubular atrophy.

Supplementary Table 5 Cox hazards model analysis of variables regarding percentage for crescents, sclerotic glomeruli, and normal glomeruli for ESKD during follow-up in patients AAV-GN

Variables	Univariable			Multivariable		
	HR	95% CI	P value	HR	95% CI	P value
Crescents (cellular+fibrocellular, %)	1.006	0.994, 1.018	0.308			
Global glomerulosclerosis (%)	1.016	1.005, 1.028	0.004	1.007	0.996, 1.019	0.217
Normal (%)	0.972	0.956, 0.988	0.001	0.975	0.958, 0.992	0.004

ESKD: end-stage kidney disease; AAV: ANCA-associated vasculitis; GN: glomerulonephritis.
